# Respiratory symptoms related to flour dust exposure are significantly high among small and medium scale flour mill workers in Ethiopia: a comparative cross-sectional survey

**DOI:** 10.1186/s12199-021-01019-y

**Published:** 2021-09-29

**Authors:** Tesfaye Hambisa Mekonnen, Awrajaw Dessie, Amensisa Hailu Tesfaye

**Affiliations:** Department of Environmental and Occupational Health and Safety, Institute of Public Health, College of Medicine and Health Sciences, University of Gondar, P.O. Box 196, Gondar, Ethiopia

**Keywords:** Work-related respiratory symptoms, Comparative cross-sectional, Flour dust, Flour mills, Ethiopia

## Abstract

**Background:**

International Labour Organization (ILO) report indicates more than 2.4 million workers die from work-related diseases and accidents each year. Work-related respiratory ailments related to airborne particulate matter such as flour dust are responsible for about 386,000 deaths and 6.6 million illness-adjusted life years. Even though exposure to flour dust together with the extreme expansions of flour mill sectors is a priority health concern, extent of the problem is little investigated in Ethiopia. The aim of this study was to evaluate the magnitude and risk factors of work-related respiratory symptoms among flour mill workers in Bahir Dar City, Ethiopia.

**Methods:**

This study employed a comparative cross-sectional survey of 560 samples (280 exposed group from flour mill workers and 280 unexposed group from office workers) with a stratified random sampling technique. The study was conducted from March to April 2019 in Bahir Dar City, Northwest Ethiopia. We used the British Medical Research Council (BMRC) questionnaire to assess work-related respiratory symptoms. The questionnaire was pretested and interview administered to collect data. Binary logistic regression analysis was fitted to evaluate significant factors of respiratory symptoms at a < 0.05 *p* value. Adjusted odds ratio (AOR) with a confidence interval (CI) of 95% was calculated to determine a strength of association.

**Results:**

All the sampled participants had fully responded to the interview. The median age of exposed and unexposed groups was 28.5 interquartile range (IQR, 20) and 31 (IQR, 15) years, respectively. The prevalence of work-related respiratory symptoms among flour mill workers was substantially higher than that of among controls, 63.9% and 20.7%, respectively (*Χ*^2^ = 107.11; *p* < 0.0001). Chest illness among flour mill workers was higher, 43.6% (*N* = 122) compared to that of among control group, 7.9% (*N* = 22) followed by dyspnea, 33.6% (*N* = 94) versus 2.5% (*N* = 7) among flour mill workers and control group, respectively. Age > 35 years [AOR, 2.03; 95% CI (1.34, 5.48), having no education [AOR, 1.54; 95% CI (1.28, 3.06)], work set up with inadequate ventilation [AOR, 2.05; 95% CI (1.18, 3.56)], work experience > 5 years [AOR, 1.89; 95% CI (1.23, 4.67)] and having no training in safety [AOR, 2.45; 95% CI (1.45, 4.76)] significantly affected the experience of respiratory symptoms among flour mill workers whereas age [AOR, 1.79; 95% CI (1.06, 3.04)], monthly salary [AOR, 1.98; 95% CI (1.04, 3.78)] and exposure status [AOR, 5.18; 95% CI (3.34, 8.04)] were detected to be significant factors of respiratory symptoms in the exposed and the unexposed combined model.

**Conclusion:**

Respiratory symptoms emanating from exposure to various flour dusts were significantly higher among flour mill workers than among the control group. Therefore, we recommend the need to effectively implement health and safety programs that account for the reduction of dust at a source, use of engineering controls (e.g., provision of adequate ventilation systems), use of administrative measures (e.g., training program and health surveillance) and provision of a suitable personal protective equipment (PPE). Furthermore, it is vital to integrate workplace health and safety programs to the wider public health policies and strategies to effectively mitigate the burden of work-related respiratory conditions. We also encourage future studies to evaluate concentration of flour dusts combined with physical examinations to establish plausible associations of respiratory symptoms with dusts of flour mill-related origin.

## Background

Recent International Labor Organization (ILO) survey demonstrates more than 2.4 million workers die from work-related illnesses and accidents annually [1]. World Health Organization (WHO) report also shows that about 2.1% of all deaths and 2.7% of all global disease burdens are attributed to occupational risks [2]. Of work-related diseases, respiratory illnesses represent 60% of all disease and injury mortalities and 70% of all occupational disease mortalities [3]. Respiratory disorders due to airborne particulate matter result in about 386,000 deaths and 6.6 million disability-adjusted life years (DALYs) worldwide [4]. Respiratory symptoms and diseases emanating from particulate matters are on the rise and demand due responses in developing countries including Africa [5]. In developing countries, workplace conditions are often inadequately arranged in safety while the use of obsolete/banned equipment with the potential to produce profoundly high specks of dusts is usually perceptible.

Flour dust from various processing industries has been recognized as one of the major causes of respiratory health effects [6–8]. Persistent exposure, even to a low level of flour dust components including enzymes, proteins, and baker’s additives may lead to non-allergic and allergic reactions [9] which in turn results in considerable impairments and decrease in pulmonary functions [10, 11]. The methods involved in milling operations including cleaning, conditioning, milling, reduction of seeds to flour, and flour dressing and packing in flour plants produce a large quantity of potentially inhalable dust [12, 13]. Constant exposure to a high concentration of such dusts above the recommended level (usually 0.5mg/m^3^ per day) for a prolonged time also leads to the development of asthma symptoms [11, 14]. Consequently, the experience of symptoms of respiratory ill-health and the decrease in lung function capacities are commonly documented among workers in flour mill industries [15].

A number of epidemiological studies documented a high prevalence of respiratory symptoms associated with exposure to flour dusts. Accordingly, the incidence varies from 15.8% in Iran [11] to 18% in Canada [16], 19% in India [14] to 90% in Egypt [8]. A recent study in Addis Ababa, Ethiopia, indicates that 27.7% of workers exposed to flour dust reported dry cough compared with 9.3% in the control group counterpart [6]. The relationships of occupational related respiratory symptoms and factors of workplaces, individual, and behavioral styles have been well established as well. Duration of exposure to high levels of flour dusts [8, 11], sex, age, education, experience, and trainings in safety [17, 18], and smoking [16, 18] are among other factors that worsen the experience of work-related respiratory symptoms.

Ethiopia is one of the largest grain producers in Africa, and the second largest wheat producer in Sub-Saharan Africa, following South Africa [19]. As a result, Ethiopia’s food production industries have been at a dramatically increasing pace over the last decade. However, the industries are also daunting for varieties of health and safety challenges. On the one hand, the sectors are often restricted to small and medium size and there is lack of modern technological manufacturing methods and financial resources to conform health and safety regulations [20]. On the other hand, because manpower is cheaper in developing countries including Ethiopia, the companies tend to generate more profits with low costs, prioritizing short-term economic gains over safety concerns [21], while many of the industries are precarious, and, often not accessible to labor supervisory staff, enforcements of safety and health standards usually remains fragile [22]. Moreover, workers in flour mill firms spend much of their working times under unhygienic conditions and poorly ventilated work set-ups [6].

In light of the scenario, as a key component of public and occupational health initiatives, it urges to produce data that help take measures to prevent workers’ exposure, and to improving safety in flour mill industries. Such data are negligible and therefore hard to infer policy in Ethiopia. This study employed a comparative cross-sectional survey of flour mill workers to estimate respiratory symptoms and their associated factors in Bahir Dar City, Northwest Ethiopia.

## Methods

### Study design and setting

This study was a comparative cross-sectional survey conducted in Bahir Dar City, Northwest Ethiopia, from March to April 2019 among flour industry workers. The city is the capital of the Amhara National Regional State, situated 540 km from Addis Ababa, the capital of Ethiopia. The estimated total population of Bahir Dar is 243,300. The main investments in the city include large- and medium-scale manufacturing industries such as textile, food and soft drink, soap production, and flour processing. Flour mills attract the larger workforce, especially the lower-wage workers in the city. In the city, there were a total of 1600 flour industry workers.

### Source and study population

The source population was all workers in flour mill factories in Bahir Dar City. Workers who had a history of exposure to different sources of dusts including silica and coal dust, in other workplaces a year before the start of the study were excluded. Besides, workers with a history of smoking and asthma or chronic obstructive pulmonary disease (COPD) prior to the start of the current job were also excluded. The source population for the comparison/control/unexposed group was office workers who had no known exposure to flour dust, smoking history, chronic respiratory and other chronic diseases, and who lived in Bahir Dar City for at least 1 year before the inception of the study.

### Sample size determination and sampling procedures

The sample size was calculated by a double population proportion formula. We employed the EpiInfo program version 7 software to estimate the sample size. Then, we employed the proportion of respiratory symptoms among exposed (flour mill workers) (35.1%), the proportion of respiratory symptoms among unexposed group (19.9%) [14] with 95% CI, 80% desired power, 5% margin of error, 10% non-response rate, and 1:1 exposure ratio to unexposed group. Finally, a total of 560 participants enrolled from the two groups: 280 from exposed and 280 from unexposed groups.

All the 12 flour mill factories in Bahir Dar City were eligible for the study. The study subjects were selected from each flour mill with a proportional allocation. Furthermore, the samples were stratified by departments, i.e., cleaning, packing, milling, and storage because we assumed there is heterogeneity in exposure to dusts in each department. After allocating participants to each stratum proportionally to their size, they were selected by a simple random sampling technique.

The unexposed group was selected from public workers in Bahir Dar City. Ten randomly selected offices were included in the study and workers from each selected office were selected by a simple random sampling method. We applied frequency matching because we equally distributed the matching variables among exposed and unexposed groups. The unexposed were matched with the exposed workers by sex, marital status, body mass index (BMI), and residence.

### Measurement of variables

Work-related respiratory symptoms, the primary outcome variable of the study, was counted if the workers had experienced the symptoms including cough, phlegm, wheezing, dyspnea, chest pain, chest tightness, and breathlessness which lasted for at least 3 months in the past year. Ventilation condition of the working units has been reported as good if the unit is equipped with functional mechanical ventilation systems (ventilator, local exhaust ventilation system) and natural ventilation systems (doors, windows, and any other openings), and it is poor if there is no functional mechanical and natural ventilation systems in the units and if the airflow is hindered by adjacent buildings and poor layout of the unit.

Body mass index (BMI) was calculated by dividing a self-report weight in kilograms by height squared in meters (kg/m^2^). Then, we operationalized as underweight = BMI < 18.50; normal = BMI 18.50–24.99; overweight/obese = BMI ≥ 25.00.

Previous exposure history shows workers’ experience in the dusty environment before the current flour mills [23]. Medium-scale industry is a flour mill that employs 26‑100 workers, whereas small-scale industry contains 5‑25 workers.

### Data collection tools and procedures

The standardized British Medical Research Council (BMRC) questionnaire was used for data collection [24]. The tool has been tested and has been used in several contexts, and is a reproducible and accurate to collect such information [17, 25]. The instrument was also used in recent studies in Ethiopia, and it was adapted to the local culture and conditions [17, 26, 27]. Overall, the detailed questionnaire we used consisted of four sections including socio-demographic, behavioral, occupational and environmental factors, and tools for assessments of respiratory symptoms.

The questionnaire was pre-tested on 5% (28 participants) in Gondar City flour mill workers before the actual survey. It was then subsequently adjusted for clarity, length, wording, and logical sequence. A face-to-face interview data collection technique was employed. Training was given to data collectors and supervisors for 2 days. Moreover, we offered briefings to the respondents regarding the aim and objectives of the study and consent to participate. In addition, constant and strict monitoring and on-the-spot reviews were carried out during data collection process and workplace observation checklist was prepared to observe the work units.

### Data processing and analysis

The data were reviewed, coded, and entered in to the epidemiological information package (EPi-info) version 7.2.0.1 program software and exported to the social sciences statistical package (SPSS) program version 20 software for further analysis. Descriptive findings were presented with frequencies and percentages for most variables. The difference in the percentage was statistically tested by the chi-square test. A bivariate logistic regression analysis was conducted primarily to pick variables for the final model based on a cut off < 0.2 *p* value. Two multivariate binary logistic regression models were fitted. The first, large model, for both exposed and non-exposed groups, was performed to determine the frequency of respiratory symptoms, to evaluate socio-demographic characteristics and personal factors. The second, reduced model for the exposed group was designed to examine predictor variables for respiratory symptoms. Next, before fitting the binary logistic regression model, the consistency of the model fit was tested by Hosmer-Lemeshow and the assumption was fulfilled (*p* value > 0.05). The significance level was obtained at a CI of 95% and ≤ 0.05 *p* value. The adjusted odds ratio (AOR) was used to determine the strength of associations.

## Results

### Socio-demographic characteristics of study participants

Response rate was 100%. More than half of the flour mill workers (155 (55.4%)) and more than half of the control (156 (55.7%)) were male. The median age of exposed and non-exposed participants was 28.5 (IQR, 20) and 31 (IQR, 15) years, respectively. About one-fourth of the exposed, 80 (28.6%) and less than half of the control, 118 (42.1%) were in the age group of 25‑35 years. About three-fourth, 219 (78.2%) of the flour mill workers, and nearly all, 274 (97.9%) of the office workers were literate. Of the participants, 225 (80.4%) and 264 (94.3%) were urban dwellers from flour mill workers and office workers, respectively (Table 1).

**Table 1:** Socio-demographic characteristics, Bahir Dar City, Ethiopia, 2019 (*N* = 560)

Characteristic	Category	Flour mill workers, ***N*** (%)	Control group, ***N*** (%)	***P*** value
**Sex**	Female	125 (44.6)	124 (44.3)	1.000
	Male	155 (55.4)	156 (55.7)	
**Age (years)**	18‑24	116 (41.4)	80 (28.6)	0.001
	25‑35	80 (28.6)	118 (42.1)	
	> 35	84 (30.0)	82 (29.3)	
**Residence**	Urban	225 (80.4)	264 (94.3)	0.38
	Rural	55 (19.6)	16 (5.7)	
**Educational status**	Cannot read and write	61 (21.8)	6 (2.1)	< 0.0001
	Can read and write	219 (78.2)	274 (97.9)	
**Marital status**	Married	151 (53.9)	146 (52.1)	0.74
	Unmarried	129 (46.1)	134 (47.9)	
**Monthly income (ETB)**	< 2000	114 (40.7)	31 (11.1)	< 0.0001
	2000‑2900	85 (30.4)	53 (18.9)	
	2901‑4050	54 (19.3)	89 (31.8)	
	> 4050	27 (9.6)	107 (38.2)	
**BMI**	Normal	235 (83.9)	237 (84.6)	0.075
	Underweight	17 (6.1)	7 (2.5)	
	Overweight/obese	28 (10.0)	36 (12.9)	
**Physical exercise**	Yes	42 (15.0)	76 (27.1)	0.001
	No	238 (85.0)	204 (72.9)	

Keys: *BMI* body mass index, *ETB* Ethiopian birr (currency), *N* number

### Workplace and behavioral characteristics of participants

The majority of the flour mill workers worked in medium-scale mills, 52.1% (*n* = 146). Less than half of the flour mill workers, 40.4% (*n* = 113), and few of the control group, 8.6% (*n* = 24) worked in shift work. With respect to ventilation in the workplaces, more than half, 55.4% (*n* = 155) and less than half, 40.4% (*n* = 113) of the flour mill workers and control group respectively, reported that they worked in the plants and offices where there was no adequate ventilation. More than one-third, 36.8% (*n* = 103) of the participants from flour mill factories and almost one-third, 31.1% (*n* = 87) of the office workers indicated they took trainings in health and safety. The majority, 142 (50.7%) and about one-third, 28.2% (*n* = 79) of the flour mill employees and office workers, respectively, worked > 8 h per day. Moreover, 86% (*n* = 241) of flour mill workers reported their working unit is cleaned by a vacuum cleaner and 80% (*n* = 224) of them reported they used personal protective equipment (PPE) on duty (Table 2).

**Table 2:** Workplace and behavioral characteristics among participants (*N* = 560)

Characteristics	Category	Flour mill workers, ***N*** (%)	Control, ***N*** (%)	***P*** value
Shift work	Yes	113 (40.4)	24 (8.6)	0.801
	No	167 (59.6)	256 (91.4)	
Ventilation	Adequate	125 (44.6)	167 (59.6)	< 0.0001
	Inadequate	155 (55.4)	113 (40.4)	
Safety training	Yes	103 (36.8)	87 (31.1)	0.001
	No	177 (63.2)	193 (68.9)	
Pre-employment medical check-up	Yes	167 (59.6)	97 (34.6)	0.310
	No	186 (66.4)	183 (65.4)	
Regular medical check-up	Yes	63 (22.5)	85 (30.4)	0.600
	No	217 (77.5)	195 (69.6)	
PPE use	Yes	56 (20.0)	76 (27.1)	0.002
	No	224 (80.0)	204 (72.9)	
Service year	<=5	167 (59.6)	79 (28.8)	< 0.0001
	>5	113 (40.4)	201 (71.8)	
Working hours per day	<=8	138 (49.3)	201 (71.8)	0.410
	>8	142 (50.7)	79 (28.8)	

Keys: *N* number; *PPE* personal protective equipment

### Prevalence of respiratory symptoms

The prevalence of respiratory symptoms among exposed (flour mills) and unexposed (office workers) was 63.9% [95% CI (58.4, 69.5)] and 20.7% [95% CI (15.9, 25.4%)], respectively. Whereas the overall prevalence rate in both exposed and unexposed groups was 42.3% (*N* = 237 [95% CI (38.6, 46.6)]. The prevalence of respiratory symptoms was significantly higher among flour mill workers than among office workers (*Χ*^2^ = 107.11; *p* value < 0.0001). The perceived symptoms reported among flour mill workers were cough (29.3%), productive cough (26.1%), phlegm (30.4%), chest illness (43.6%), and dyspnea (33.6%) (Table 3).

**Table 3:** Prevalence of respiratory symptoms among exposed and unexposed groups (*N* = 560)

Work-related respiratory Symptoms	Flour mill workers, ***N*** (%)	Control group, ***N*** (%)	***P*** value
Cough	82 (29.3%)	23 (8.2%)	< 0.0001
Productive cough	73 (26.1%)	5 (1.8%)	< 0.0001
Phlegm	85 (30.4%)	30 (10.7%)	< 0.0001
Chest illness	122 (43.6%)	22 (7.9%)	< 0.0001
Dyspnea	94 (33.6%)	7 (2.5%)	< 0.0001

Keys: *N* number

### Findings from observations

Based on walk through observations of the flour mill plants, we found that there were poor technological standards used to control generation of air-borne particulate matter. We have noticed that poor indoor air quality is predominating in the workplace. Poor housekeeping procedures were detected in all working departments. Although openings (entryways, windows, and other openings) were available, the wind stream in various working units was obstructed because of poor workstations, poor plan, and design of the machines and working units. We also noticed that each working department did not have adequate illuminations. There has been no safety communication in the majority of department in the premises. None of the plants rehearsed sodden wiping to restrict the introduction of flour dusts. There were no basic facilities such as washing facilities at many of the observed plants. The majority of workers are not provided with appropriate PPE and those few workers who were provided with PPE did not wear it, handled it unsafely, and placed it somewhere at their worksite. On the other hand, where PPE is available, it is not a standard; it has been teared and placed by the workers and taken to their homes as there is no supervisory mechanism for its use. In general, the observation confirmed that the working environment of the flour mills might justify the higher proportion of the respiratory symptoms reported in the study.

### Factors associated with work-related respiratory symptoms

Two models were fitted to determine the associated factors of respiratory symptoms; one large model for the entire data set, and the second for the flour mill workers. In the former model, the effect of exposure status on respiratory symptoms was determined after adjusting confounding factors, like sex, age, educational status, monthly income, physical exercise, and BMI. The result indicated that the odds of respiratory symptoms were 5.18 times higher among flour mill workers than among the control (AOR = 5.18; 95% CI, 3.34, 8.04). Age and monthly income were also associated with respiratory symptoms in the larger model (combination of exposed and unexposed group) (Table 4).

**Table 4:** Factors associated with respiratory symptoms among exposed and unexposed groups, Ethiopia, 2019

Characteristic (***N*** = 560)	Respiratory symptoms		COR (95% CI)	AOR (95% CI)
	Yes	No		
**Exposure status**				
Flour mill workers	179	101	6.78 (4.65, 9.90)	5.18 (3.34, 8.04)
Controls	58	222	1	1
**Sex**				
Female	105	144	0.99 (0.71, 1.39)	0.94 (0.61, 1.43)
Male	132	179	1	1
**Age**				
18‑24	87	109	1	1
25‑35	67	131	0.64 (0.43, 0.96)	0.96 (0.59, 1.57)
> 35	83	83	1.25 (0.83, 1.89)	1.79 (1.06, 3.04)
**Educational status**				
Illiterate	47	20	3.75 (2.15, 6.52)	1.77 (0.96, 3.25)
Literate	190	303	1	1
**Marital status**				
Married			1	1
Unmarried			0.86 (0.61, 1.20)	0.83 (0.55, 1.25)
**Monthly income (ETB)**				
< 2000	86	59	3.82 (2.31, 6.32)	1.98 (1.04, 3.78)
2000‑2900	69	69	2.62 (1.58, 4.34)	1.64 (0.89, 3.01)
2901‑4050	45	98	1.20 (0.72, 2.02)	1.01 (0.56, 1.82)
> 4050	37	97	1	1
**Physical exercise**				
Yes	43	75	1	1
No	194	248	1.36 (0.89, 2.08)	0.90 (0.55, 1.49)
**BMI**				
Normal	199	273	1	1
Underweight	10	14	0.98 (0.43, 2.25)	0.63 (0.25, 1.61)
Overweight/obese	28	36	1.07 (0.63, 1.80)	1.29 (0.71, 2.35)

Key: *BMI* body mass index, *ETB* Ethiopian birr, *COR* crude odds ratio, *AOR* adjusted odds ratio

In the second model (the model consisting only flour mill workers), age, educational status, status of working unit ventilation, year of service, and health and safety trainings were significantly associated with respiratory symptoms (Table 5).

**Table 5:** Factors associated with respiratory symptoms among flour mill workers, Ethiopia

Characteristics (*n* = 280)	Respiratory symptoms		COR (95% CI)	AOR (95% CI)
	Yes	No		
**Age**				
18‑24	70	46	1	1
25‑35	44	36	0.80 (0.45, 1.43)	0.69 (0.32, 1.12)
> 35	65	19	2.25 (1.19, 4.23)	2.03 (1.34, 5.48)
**Monthly income (ETB)**				
< 2000	78	36	1.49 (0.63, 3.53)	1.08 (0.54, 4.84)
2000‑2900	55	30	1.26 (0.52, 3.06)	1.02 (0.38, 3.52)
2901‑4050	30	24	0.86 (0.34, 2.19)	0.72 (0.21, 2.74)
> 4050	16	11	1	1
**Educational status**				
Cannot read and write	51	46	2.10 (1.48, 3.67)	1.54 (1.28, 3.06)
Can read and write	128	55	1	1
**Ventilation**				
Adequate	67	58	1	1
Inadequate	112	43	2.25 (1.37, 3.71)	2.05 (1.18, 3.56)
**Cleaning the room by vacuum cleaner**				
Yes	15	24	1	1
No	164	77	3.41 (1.69, 6.86)	2.51 (0.91, 5.49)
**Work experience in years**				
≤ 5	90	77	1	1
5+	89	24	3.17 (2.34, 5.25)	1.89 (1.23, 4.67)
**Regular medical check-up**				
Yes	34	29	1	1
No	145	72	1.72 (0.97, 3.04)	1.80 (0.93, 3.46)
**Health and safety training**				
Yes	54	59	1	
No	125	42	3.25 (1.86, 6.45)	2.45 (1.45, 4.76)
**Working hours/day**				
≤8	73	65	1	
> 8	105	37	2.53 (1.8, 3.98)	1.84 (0.82, 4.34)

Keys: *AOR* adjusted odds ratio, *CI* confidence intervals, *COR* crude odds ratio, *ETB* Ethiopian birr (currency), *n* number

The current study showed that age was significantly associated with respiratory symptoms among flour mill employees. Workers with > 35 years of age were 2.03 more likely to develop respiratory symptoms [AOR = 2.03; 95% CI (1.34, 5.48)]. The chance of developing respiratory symptoms among flour mill workers who had no education were 54% higher than those of the literate workers [AOR = 1.54; 95% CI (1.28, 3.06)]. Workers who worked in an inadequately ventilated unit had 2.05 times higher at risk to experience respiratory symptoms than those who operate in well-ventilated working units [AOR = 2, 05; 95% CI (1. 18, 3. 56)]. Workers with more than 3 years of services/work experience had 89% increased chance of developing respiratory symptoms than those with less than 3 years of services [AOR = 1.89; 95% CI (1.23, 4.67)]. The probability of developing respiratory symptoms is increased by a factor of 2.45 among workers who did not receive trainings in health and safety than those who received the training (AOR = 2.45, 95% CI, 1.45, 4.76) (Table 5).

## Discussion

Small- and medium-size manufacturing industries have been growing over the past decade in Ethiopia. Together with the intensive expansion of the industries, workers’ exposures to various traditional and newly emerging hazards are increasing considerably. Efforts to curb such risk exposures in these industries, however, was conferred the least priority. In this study, we conducted a comparative cross-sectional survey of flour mill workers to analyze the magnitude of respiratory symptoms and its associated factors in Northwest Ethiopia. The result of the study reveals that a higher proportion of respiratory symptoms among flour mill workers were observed than among the control group (63.9% vs. 20.7%). Workers in flour mill factories are more likely subject to exposure to a high level of flour dusts for a prolonged period. The report by scholars in Sudan [28] and Italy [29] concords this finding. Dust containing various contaminants including silica, bacterial endotoxins, pollen, fragments of insects, mites, animal dander, bird and rodent feces, and other chemical additives such as pesticides and herbicides has the potential to increase respiratory dysfunctions [8, 30]. Findings in Iran [31], Nigeria [32], and Macedonia [33] noted that there is a substantial difference in incidence rate of respiratory symptoms between workers exposed to flour mill dusts and not exposed to flour dusts (unexposed group).

Lower prevalence rate of respiratory symptoms has been documented among flour mill workers in studies in Ethiopia (Hawassa) (56.6%) [34], Iran (28%) [31], the UK (22%) [35], Nigeria (49.5 and 54%) [32, 36], and Macedonia (41.8%) [33] compared to the current finding. The possible reason might be attributed to variations in the levels of health and safety implementation, method of collecting data (self-report versus clinical diagnosis), effective use of PPE, and response rate. On the other hand, a higher prevalence of respiratory symptoms than our finding has been reported in Egypt (90%) [8]. This could be because of the differences in data collection technique, prevalence of other concomitant symptoms and data collection period.

Our analysis revealed that older workers in flour mill industries are more likely than their younger counterparts to suffer from respiratory symptoms. The overall model (both exposed and unexposed) has also detected that age is a significant predictor of respiratory symptoms but not the model in unexposed group. This finding is consistent with other reports [8, 37]. Aging is the process likely linked to chronic and disabling diseases, which may be the possible reason for this finding. Furthermore, as age increases, defense mechanisms of the body will decline and vulnerability to health risks likely increase, which may be the other possible explanation.

In the current survey, flour mill workers with no education reported higher respiratory impairments than educated participants. This report replicates findings of numerous epidemiological studies [17, 34, 38, 39]. This explains that attaining some form of education would likely advance employees’ knowledge about workplace health risks and bequeaths mechanisms to prevent and control workplace hazards. Moreover, safety communication as a means of risk reduction is also a major concern in Ethiopia, as the majority of the workers in small- and medium-scale industries are those who cannot read and write.

Similar to our finding, multiple studies have proven that the length of employment substantially affects the likely occurrence of respiratory symptom [8, 33, 40–42]. The probable suggestion may be that as the duration of employment increases, exposure to potential predisposing factors will increase, which in turn elevates the accumulation of inhalable hazards in the respiratory systems. Moreover, this could be explained in that work-related respiratory conditions can have long latency periods, and are more noticeable years after employment stay.

The lack of adequately ventilated working units was the other important factors influencing the incidence of respiratory conditions among flour mill workers. Previous evidence has registered a reliable finding [43]. The levels of contaminants and pollutants in a poorly ventilated working environment may increase with a potential increase in exposure for employees. This is also supported by findings from workplace observations in which wind streams were obstructed in almost all of the observed working units because of poor workstations, poor plan, and design of the machines and working units, poor housekeeping procedures, and poor indoor air quality.

The lack of ventilation also may lead to the accumulation of flour dust in the indoor environment and decreases the mixing and dilution of those dusts [8, 25, 26, 28, 44]. Ethiopia is one of the developing countries where the majority of workplace conditions and facilities are often inadequately equipped. There are some labor proclamations and regulations that allow employers to ensure that the work place and premises do not cause danger to the health and safety of the workers. In majority of small- and medium-size industries, however, this is often less pragmatic due to the insecure nature of those industries. Therefore, workers, to cover their daily breads, are compelled to attend their duties under harsh working conditions, which in turn worsen the incidence of respiratory health conditions.

Our analysis revealed that the lack of trainings in safety and health of the workplaces considerably affected the occurrence of work-related respiratory problems. Evidence in the literature has confirmed our result [17, 26]. Safety and health trainings could likely boost the awareness, knowledge, and skills of the workers to prevent and control hazards and risks at their workplaces. Trainings in safety may also positively influence the behavior of employees to improve safety cultures and comply with workplace regulations and standards. In addition, basic components of trainings in safety such as the identification, recognition, and evaluation of hazards and risks as well as measures for emergency situations would help reduce the potential health effects emerging from workplaces [45, 46].

Similar to every other developing country, workers in small- and medium-size industries are usually least considered in health and safety regulations in Ethiopia. The health conditions that the workers often encounter have been little investigated in such industries. Therefore, there has been a shortage of reliable data that help develop preventive measures, which commensurate with the risks emerging alongside with the extensive growth of the industries. We believe that the findings of this survey cannot be overlooked as it minimizes those gaps. We also employed a comparative cross-sectional design to support the hypothesis that exposure to the inherent nature of hazards in flour mill industries is substantially linked to the experience of work-related respiratory conditions. Besides, the interview data collection technique used in the current investigation has also increased the response rate, which in turn ensures that the findings are representative.

However, there are some limitations which cannot be revoked in the current investigation. First, because of the feasibility concern, measurements for the lung function and other examinations such as prick tests were not carried out. Second, the study failed to determine the levels and characteristics of flour dust exposures. Third, there was a lack of information on the types of energy the participants used at their homes. Fourth, the study was carried out in specific types of workplaces (flour mill factories), which precludes the findings from being expanded to other economic sectors. Moreover, because the unexposed group participants were selected from public workers, they might differ from the flour mill workers in many characteristics. Finally, because of a recall bias, underreporting may be anticipated, as the study relied on self-reporting data. We fitted separate models to minimize the effects of some confounders, and used a validated assessment tool to evaluate the symptoms.

## Conclusion

Respiratory symptoms related to occupational exposure to flour dusts were significantly higher among flour mill workers than those of control group. Older age, lower level of education, higher length of employment, lack of trainings in safety and health, and inadequate ventilation in the workplaces were important factors of work-related respiratory impairments. We recommend that employers need to effectively implement health and safety programs that account for the reduction of dusts at source, the use of engineering controls (e.g., provision of adequate ventilation systems), the use of administrative measures (e.g., training program and health surveillance), and provision of suitable personal protective equipment (PPE) in the workplace. Moreover, aligning workplace health and safety programs with wider public health policies and strategies to effectively mitigate the burden of respiratory conditions is recommended. We also encourage future studies to evaluate concentration of flour dusts combined with physical examinations to establish plausible associations of respiratory symptoms with dusts of flour mill-related origin.

## Data Availability

All data generated or analyzed during this study are included in this article. The data that support the findings of this study are also available from the corresponding author upon a reasonable request.

## Abbreviations

AOR: Adjusted odds ratio
BMI: Body mass index
BMRC: British Medical Research Council
CI: Confidence interval
COPD: Chronic obstructive pulmonary disease
COR: Crude odds ratio
DALYs: Disability-adjusted life years
IQR: Inter quartile range
SPSS: Statistical Package for Social Science
UK: United Kingdom
